# Gene Expression Order Attributed to Genome Reduction and the Steady Cellular State in *Escherichia coli*

**DOI:** 10.3389/fmicb.2018.02255

**Published:** 2018-09-20

**Authors:** Bei-Wen Ying, Kazuma Yama

**Affiliations:** ^1^Institute of Biology and Information Science, East China Normal University, Shanghai, China; ^2^Faculty of Life and Environmental Sciences, University of Tsukuba, Tsukuba, Japan; ^3^Advanced Analytical Science Laboratories, Research & Development Headquarters, Lion Corporation, Tokyo, Japan

**Keywords:** transcriptome, power law, gene expression, genome reduction, growth rate, cellular state, *Escherichia coli*

## Abstract

Transcriptomes not only reflect the growth status but also link to the genome in bacteria. To investigate if and how genome or cellular state changes contribute to the gene expression order, the growth profile-associated transcriptomes of an assortment of genetically differentiated *Escherichia coli* either exponentially growing under varied conditions or in response to environmental disturbance were analyzed. A total of 168 microarray data sets representing 56 transcriptome variations, were categorized by genome size (full length or reduced) and cellular state (steady or unsteady). At the genome-wide level, the power-law distribution of gene expression was found to be significantly disturbed by the genome size but not the cellular state. At the regulatory network level, more networks with improved coordination of growth rates were observed in genome reduction than at the steady state. At the single-gene level, both genome reduction and steady state increased the correlation of gene expression to growth rate, but the enriched gene categories with improved correlations were different. These findings not only illustrate the order of gene expression attributed to genome reduction and steady cellular state but also indicate that the accessory sequences acquired during genome evolution largely participated in the coordination of transcriptomes to growth fitness.

## Introduction

Genome reduction performed in bacterial cells is a practical approach not only to study the essential gene set of a living cell but also to estimate the potential contribution of redundant genomic sequences. An assortment of reduced genomes has been constructed in *Escherichia coli* (Posfai et al., 2006; Kato and Hashimoto, 2007; Mizoguchi et al., 2008; Karcagi et al., 2016) and *Bacillus subtilis* (Morimoto et al., 2008; Reuss et al., 2017). In addition to these achievements in genetic engineering, multilevel evaluations of the effect of genome reduction to cell life (Karcagi et al., 2016; Kurokawa et al., 2016; Nishimura et al., 2017) are performed because characterization of reduced genomes can help researchers achieve the potential function of deleted genome sequences (Karcagi et al., 2016). In particular, those deleted sequences, e.g., phages/IS in common, are supposedly largely acquired by horizontal gene transfer (HGT) during genome evolution (Dougan et al., 2001; Warnefors et al., 2010; Pal and Papp, 2013), indicating that genome reduction is a reverse process to HGT (Kurokawa et al., 2016). Although the mechanisms of HGT have been intensively studied in both prokaryotic and eukaryotic organisms during the past decades (Kurland et al., 2003; Boto, 2010), a direct and global comparison between the genomes of full length and reduced size in the same species (e.g., *E. coli*) would provide novel insights compared to the studies either focusing on specific genes horizontally transferred or comparing genomes of different species.

To acquire an overview of the cellular state, analysis of the genome-wide gene expression is one of the representative approaches (Passalacqua et al., 2009; Elliott, 2014). Previous studies have reported the differentiation in gene expression triggered by either genome reduction (Karcagi et al., 2016; Reuss et al., 2017) or changes in cellular states (Durfee et al., 2008; Jozefczuk et al., 2010). Thus far, only a few common phenomena have been reported in a global view of transcriptomes. The power law, a well-known theory of complex networks (Barabasi and Albert, 1999), has been experimentally demonstrated in gene expression across species (Ueda et al., 2004). This universal phenomenon reflects the static gene expression order at the genomic level. In addition, quantitative evaluation of transcriptomes linking to growth, which is a global parameter representing the activity and/or fitness of living cells, has demonstrated the correlations of gene expression to growth rate in *E. coli* of both the wild type genomes (full length) (Grondin et al., 2007; Nahku et al., 2010) and the reduced genome (Matsumoto et al., 2013). These studies relied on either a single genotype under diverse growth conditions or a number of genotypes or species in a defined condition. Thus, the difference and/or similarity in the order of gene expression between genome size and cellular state is under investigation.

It remains unclear if and how genome reduction and cellular state interrupt the order of global gene expression. To address this question, we analyzed 168 growth profile-associated microarray data sets of an assortment of *E. coli* strains, and we investigated the common properties of the gene expression order at genome-wide, regulatory network and single-gene levels. The power-law distribution of gene expression and the growth rate-correlated expression of both single genes and transcriptional networks were examined. The multilevel analyses were performed in a comparative manner, i.e., genome size (full length or reduced) vs. cellular state (steady or unsteady).

## Results

### *E. coli* Transcriptomes Categorized by Genome Size and Cellular State

**FIGURE 1:**
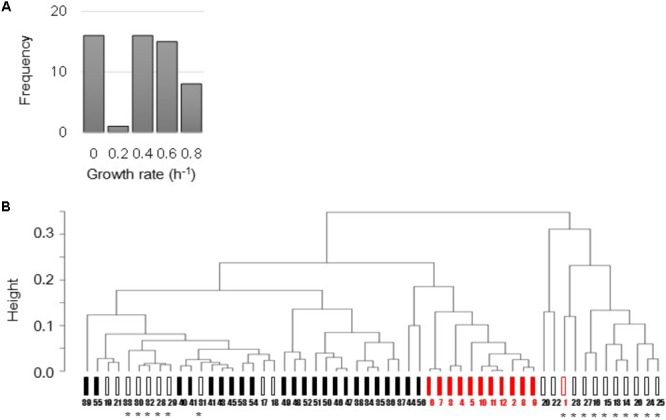
Total 56 transcriptomes used in the present study. **(A)** Histogram of 56 mean growth rates. The mean growth rates of the corresponding transcriptomes were calculated according to the previous studies. **(B)** Representative cluster dendrogram of 56 transcriptomes. Clustering analysis of gene expression was performed using the furthest neighbor method (complete mode). Filled and open bars represent the steady (exponentially growing states) and unsteady (responsive or intermediate states) cellular states, respectively. Black and red indicate the full length and reduced genomes, respectively. Asterisks indicate the unsteady states of zero growth. Numbers 1–56 correspond to 56 variations described in **Supplementary Table S1**.

The *E. coli* microarray data sets that varied in strains, culture conditions, and growth status were collected from previously published studies (Matsumoto et al., 2013; Murakami et al., 2015; Yama et al., 2015; Ying et al., 2015, 2016). Total 168 microarray data sets, which were all accompanied by the precise growth information, e.g., growth rates, were used in the present study. Following data mining, normalization and average of biological repeats, as described in the Section “Materials and Methods,” a total of 56 various transcriptomes were finally determined and associated with the mean growth rates (**Figure 1A**). These transcriptomes were manually categorized based on the genome size and the cellular state. That is, the full length (FL, 44 variations) and reduced (12 variations) genomes, and the steady (34 variations) and unsteady (22 variations) cellular states (**Figure 1B**). The details of the data sets, experimental conditions, and the analytical results were summarized in **Supplementary Table S1**.

Common clustering analysis showed that all 56 transcriptomes were roughly in the order of the differentiation in cellular states and genome size (**Figure 1B**) independent of the analytical methods (**Supplementary Figure S1**). It demonstrated that the manual categorization of the transcriptomes based on genome size and cellular states was reasonable. In addition, the transcriptomes of the steady cellular states showed high similarity regardless of genome size (**Figure 1B**, filled bars). It indicated that the genome reduction did not alter the regulatory mechanisms of gene expression that determined cellular states.

### Power-Law Distributions of Gene Expression

To achieve an overall insight of the gene expression order attributed to genome size and cellular states, the power-law distributions of gene expression were evaluated. The power law, also called *Zipf*’s law (Furusawa and Kaneko, 2003), is supposed to be a universal principle that governs the global gene expression in actively growing cells across species (Ueda et al., 2004), and thus, we investigated if this law correlated to the cellular state or genome reduction. Here, we used the cumulative probability to normalize the noise that often occurred at the large *K* of low *P(K)* as previously reported (Keller, 2005; Yachie et al., 2009). Fifty-six transcriptomes were subjected to the analysis according to the following equation:

(1) $$\begin{array}{c} P\;>\;(K)\sim K^{-r} \end{array}$$

**FIGURE 2:**
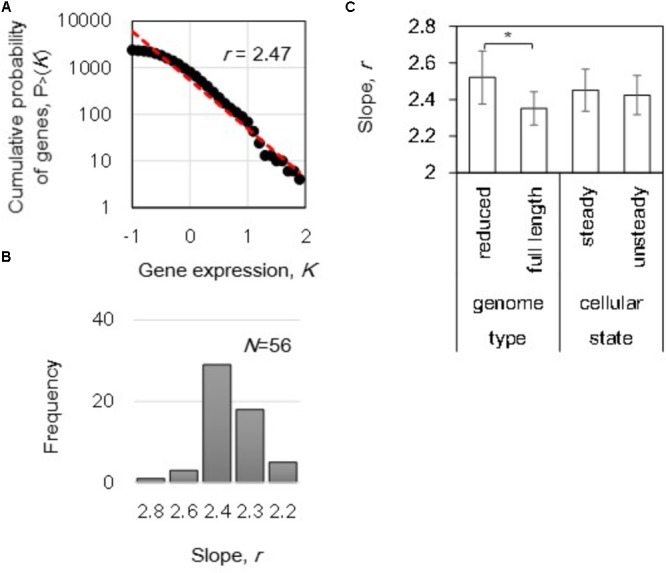
The power law in gene expression. **(A)** Cumulative probability distribution of gene expression. A power-law distribution in which the cumulative probability *P* > (*K*) that a gene has an expression level *K* is shown as an example. The red broken line indicates the regression of the decays as a power-law *P* > (*K*)∼*K^-r^* distribution. The slope of *r* is indicated. **(B)** Histogram of the slopes. The slopes (*r*) of 56 distributions (transcriptomes) were calculated and summarized in a histogram with a bin of 0.2. **(C)** Differentiated slopes in reduced genomes. The slopes of 56 distributions were averaged according to the categories of either genome size (full length and reduced) or cellular state (steady and unsteady), respectively. Statistical significance is indicated by an asterisk (*p* < 0.05).

The gene expression level in logarithmic scale was plotted against the cumulative frequency, and the slope of *r* was calculated by regression toward the distribution (**Figure 2A**). A distribution of *r*, varied from 2.2 to 2.8, was acquired from the total 56 transcriptomes (**Figure 2B**). Intriguingly, differentiation in the mean *r* of the power-law distributions in gene expression was detected in the reduced genomes (*p* < 0.05) even though the mean values of the both steady and unsteady cellular states remained equivalent (**Figure 2C**). Although gene expression dynamics follow the power law commonly from *E. coli* to *Homo sapiens* (Ueda et al., 2004), the changes in the slope *r* within an identical species was first identified in the present study. This finding strongly suggested that the gene expression orders were highly linked to the genome length and/or sequence.

In addition, the correlation of the power-law distribution to the growth rate was evaluated because transcriptome reorganization is linked to growth fitness in *E. coli* (Nahku et al., 2010; Matsumoto et al., 2013; Ying et al., 2016). However, no correlations were determined between the growth rates and the slopes (*p* > 0.1). These data indicated that the power-law distribution was independent of growth rate, which was consistent with the similar slopes found for both steady and unsteady cellular states (**Figure 2C**). Because no significant fitness-coordinated changes were detected at the genome-wide level, the growth rate-correlated expression at the individual gene and transcriptional network levels were subsequently investigated. Note that the changes in the slope *r* of reduced genomes remained significant (**Supplementary Figure S2**), even the analysis was performed without the 16 transcriptomes of zero-growth rate (**Figure 1A**).

### Improved Correlations Between Growth and Expression at Individual Gene Levels

**FIGURE 3:**
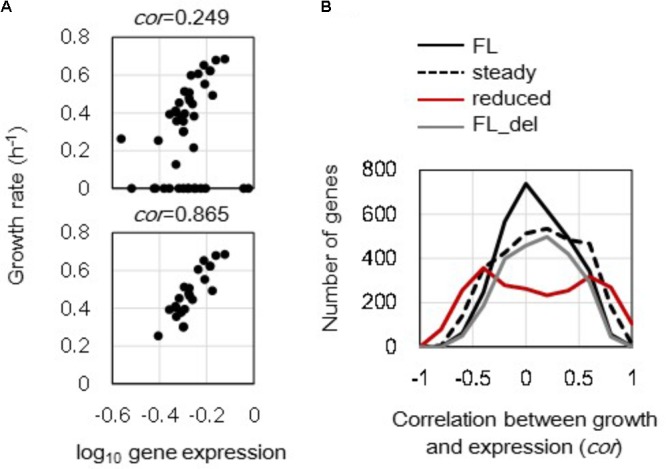
Distribution of the correlation coefficients between gene expression and growth rate. **(A)** Correlation between gene expression level and growth rate. The expression levels (mRNA concentrations in log scale) of a single gene in varied conditions were plotted against the growth rates of the corresponding conditions. The upper and bottom panels show all 44 conditions and 23 steady state conditions of *mrdB* (JW0629) in the full length genomes. The correlation coefficients are indicated. **(B)** Changes in distributions of the correlation coefficients. A total of 3213 and 2415 correlation coefficients were calculated in the full length and reduced genomes as shown in **(A)**. The numbers of the genes were counted within a bin of 0.1 for the correlation coefficient. Black solid, broken, and red lines indicate the distributions formed by 44 full length genomes, 23 steady growth of full length genome, and 12 reduced genomes, respectively. The gray line stands for the distribution formed by 44 full length genomes, in which the genes deleted in the reduced genomes were excluded from the analysis.

Changes in growth rate-correlated expression triggered by either genome reduction or steady state at the single gene level were evaluated. Correlations between the expression levels of individual genes and the corresponding growth rates (in **Supplementary Table S1**) were calculated, and the changes in correlation coefficients caused by genome reduction and cellular state were further evaluated. The correlations of *mrdB* (JW0629) in full length genomes between expression levels and growth rates were shown as an example (**Figure 3A**). The correlation coefficients between the gene expression levels and the corresponding growth rates in all (**Figure 3A**, upper) was varied from that in only 23 steady states (**Figure 3A**, bottom). All 3213 and 2415 genes in full length and reduced genomes were evaluated, and the distributions of their correlation coefficients were formed (**Figure 3B**). The full length genomes of all 44 transcriptomes formed a single peak distribution (**Figure 3B**, black solid line). It indicated that the order of growth rate-correlated expression showed higher correlation but with fewer genes. In addition, the distribution of correlation coefficients remained as a single peak but became broader when the unsteady cellular states were excluded (**Figure 3B**, broken line). That is, the correlation between gene expression and growth rate was more significant in the steady cellular state, i.e., the exponential growth phase. The broad distribution formed by the steady cellular states suggested an increased number of genes with better correlations between expression and growth rate as well as a decreased number of genes with poor correlations. In addition, the analysis was also conducted toward the data sets without the unsteady states of zero growth. The results showed that the distributions of correlation coefficients of the steady states turned similar to that of the full length, although the correlations of individual genes altered (**Supplementary Figure S3**). It implied that the differentiated distribution (**Figure 3B**, broken line) was largely caused by those of zero growth.

Interestingly, the reduced genomes showed a bimodal distribution (**Figure 3B**, red line) compared to the full length genomes that showed single peak distributions. Removal of the disappearing genes in the reduced genomes from the analysis did not alter the shape of the distribution as a single peak (**Figure 3B**, gray line). The change of the distribution from monomodal to bimodal demonstrated that the number of the growth rate-coordinated genes was increased due to genome reduction. The result indicated that the deletion of redundant sequences improved the order of gene expression in a fitness-coordinated manner. The directional change of the distribution from narrow to broad was commonly observed in the reduced genomes and steady cellular states.

### Differentiation in Gene Categories Enriched the Genes of Improved Correlations

**FIGURE 4:**
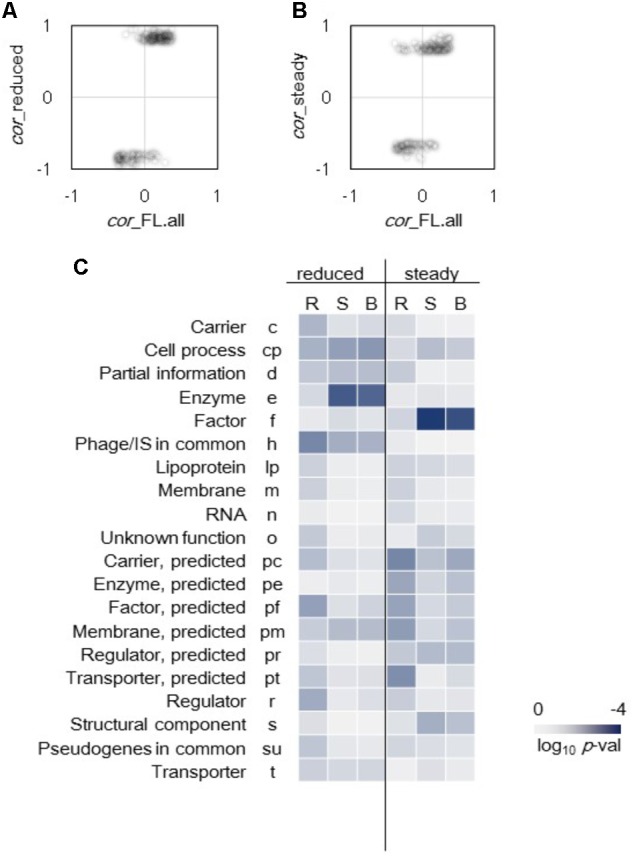
Enriched gene categories of improved correlations. **(A)** Genes of improved correlations due to genome reduction. A total of 146 genes randomly expressed (*p* > 0.01) in full length genomes changed to growth rate-coordinated expression (*p* < 0.001) in reduced genomes. **(B)** Genes of improved correlations mediated by the steady cellular state. A total of 130 genes randomly expressed (*p* > 0.01) in all 44 conditions changed to growth rate-coordinated expression (*p* < 0.001) in 23 steady growth conditions. **(C)** Heat map of gene function enrichment. Gene categories significantly contributed to the improved correlations were analyzed, and 146 and 130 genes identified in **(A)** and **(B)**, respectively, were divided into 20 gene categories. Reduced and steady indicate the changes triggered by the genome reduction and steady cellular state, respectively. R, S, and B represent the directions of the changes in correlation, namely reverse, same and both (reverse and same), respectively. Gradation from dark blue to light gray indicates the statistical significance in log-scaled *p*-values obtained using binomial tests.

Differentiation in gene categories of improved correlations to growth triggered by genome reduction and steady cellular state was observed (**Figure 4**). The genes that exhibited highly significant improved correlations between expression level and growth rate were first identified. A total of 146 genes of which expression levels had no correlation to growth in the full length (FL) genomes (*p* > 0.01) but presented high correlations to growth in the reduced genomes (*p* < 0.001) were determined (**Figure 4A**). In addition, 130 genes of which expression levels had no correlation to growth in all conditions (*p* > 0.01) but presented high correlations to growth in steady cellular states (*p* < 0.001) were determined (**Figure 4B**). Subsequently, these genes were divided into 20 gene categories (Riley et al., 2006) and were subjected to enrichment analysis. Genes with improved correlations between expression and growth were significantly enriched in the gene categories of Enzyme and Factor in the genome reduction and steady state (*p* < 0.01), respectively (**Figure 4C**). Such differentiation in gene function enrichment indicated that the genome reduction and the steady cellular state re-ordered the expression of the genes responsible for the enzymatic reactions and the molecular interactions, respectively.

The improvement of the correlations favored the same direction, that is, either an increase in positive correlation or a decrease in negative correlation (**Figures 4A,B**). Only 22 and 25 out of a total of 146 and 130 genes showing reverse directional changes in correlations were determined in the genome reduction and steady state, respectively. Such directional tendency remained even those transcriptomes of zero growth were removed from the analysis (**Supplementary Figure S4**). The reduced genome remained more on the same direction in growth correlation because fewer genes in the genome reduction showed reverse directional changes in correlation compared to those in the steady state. Enrichment analysis also showed that the gene categories of improved correlations had the same directional changes (**Figure 4C**, S and B). Taken together, genome reduction and steady cellular state favored the same directional change in gene expression, but the changes varied in gene functions. Thus, the genome reduction was linked to metabolic changes, whereas the cellular state was linked to the factors.

### Improved Correlations Between Growth and Expression at the Regulatory Network Levels

**FIGURE 5:**
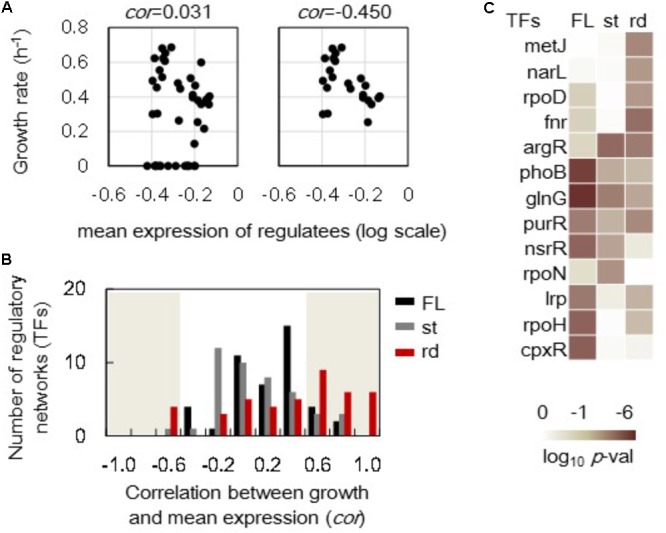
Correlations between the growth rates and the mean expression of regulatory networks. **(A)** An example of correlations between the growth rate and the mean expression of the regulatees. The genes under the regulation of *rpoS* are shown. A total of 227 and 195 genes (regulatees) were assigned under the regulation of *rpoS* in the full length and reduced genomes, respectively. The upper and bottom panels include all 44 conditions and 22 steady growth conditions in full length genomes. The correlation coefficients are indicated. **(B)** Histograms of the correlation coefficients. A total of 44 and 42 correlation coefficients (regulatory networks) were evaluated in the full length and reduced genomes as shown in **(A)**. The numbers of the regulatory networks were counted within a bin of 0.2 for the correlation coefficient. Black, gray, and red bars indicate the distributions formed by 44 full length genomes (FL), 23 steady growth of full length genome (st), and 12 reduced genomes (rd), respectively. The shadowed region indicates the statistical significance (*p* < 0.05). **(C)** Significance of correlation coefficients. Regulatory networks in which the mean expression of regulatees showed significant correlations (*p* < 0.05) to the growth rates are indicated. FL, st, and rd represent the correlation coefficients in a total 44 full length genomes, 23 steady cellular states of full length genomes and 12 reduced genomes, respectively. Gradation from dark to light indicates the statistical significance of the correlation coefficients in log-scaled *p*-values.

In addition to the growth-correlated expression of individual genes, the growth-correlated expression of regulatory networks was further analyzed. The expression levels of the genes regulated by the assigned regulator (transcriptional factor, TF) were averaged and defined as the mean expression of this transcriptional network. For example, the mean expression levels of 227 downstream genes (regulatees) under *rpoS*’s regulation in the full length (FL) genomes showed no correlation to the growth rates (**Figure 5A**, left). However, these regulatees presented a better but statistically insignificant correlation to the growth rates in steady cellular states (**Figure 5A**, right). A total of 44 and 42 TFs (transcriptional networks) that comprised more than 15 regulatees in the full length and reduced genomes, respectively, were subjected to the correlation analysis. The histograms of these 44 and 42 correlation coefficients showed there were more regulatory networks in the reduced genomes (rd) than in the full length genomes that presented better correlation of network expression to growth (**Figure 5B**, red). However, the increase in the number of the regulatory networks of improved correlations to growth rate was not detected in the steady (st) cellular state (**Figure 5B**, gray). Note that the results from the data sets excluding the zero growth remained the same (**Supplementary Figure S5**).

The regulatory networks showing the improved correlations to growth rates due to either genome reduction (rd) or steady states (st) were identified (**Figure 5C**) according to the statistical significance of the correlation coefficients of all regulatory networks (**Supplementary Figure S6**). The transcriptional network regulated by *argR* presented increased correlation coefficients between the growth rate and the mean expression of the regulatees (**Figure 5C**). It was the only regulatory network significantly influenced by genome reduction and steady state. Additionally, more transcriptional networks were solely influenced by genome reduction (e.g., *metJ, narL, rpoD*, and *fnr*) than that by steady state (e.g., *rpoN*). These results indicated that the priority of re-ordering gene expression at network levels was varied in genome reduction and steady state.

## Discussion

The present study provides a global view on the order in gene expression of genome reduction and steady cellular state. Genome size-dependent changes in the power-law distribution of gene expression (**Figure 2**) have been initially reported in the present study. These results strongly suggested that the deleted genomic sequences, which might be acquired by HGT during genome evolution (Piskurek and Jackson, 2012; Hayes et al., 2013), contributed to gene expression dynamics although these disappeared genomic sequences were redundant (Fontana and Wrobel, 2012). This finding was consistent with the previous report on the fitness decrease caused by genome reduction in other *E. coli* strains (Kurokawa et al., 2016). The increased *r* of the power-law distribution in reduced genomes suggested that the variation in expression level was less among the genes compared to that in the full length genomes. It may have been attributed to the deletion of accessory genes in the wild type genome, thus resulting in a large variation in expression level. In addition, genome reduction-induced differentiation in the distributions of the correlation coefficients (**Figure 3**) revealed that the deletion of the redundant and/or accessary sequences improved the growth-coordinated changes in gene expression. Such improved order of gene expression agreed with the previous finding demonstrating that the chromosomal periodicity is fixed in the reduced genome but flexible in the wild type genome (Ying et al., 2013). Incorporating the foreign sequences to elongate the genome length during evolution, i.e., HGT, offered the transcriptomes a better plasticity or responsivity to efficiently achieve an adaptive state in response to both intrinsic and environmental fluctuations (Soucy et al., 2015; Karcagi et al., 2016).

Correlation analyses implied that the growth fitness-coordinated transcriptome reorganization was related more to the expression of upstream modulators or regulators (e.g., Factors) at single gene levels in steady cellular states. Besides, it shaped more by the expression of downstream regulatees (e.g., Enzymes) at network levels in genome reduction (**Figures 4, 5**). The genes of significant changes in correlations were enriched in the categories of Factor and Enzyme (**Figure 4C**). For instance, the genes of *rseA* and *yefM*, which were enriched in Factor (f), encoded an anti-sigma factor and an antitoxin, respectively. It suggested that the genes play an essential role in growth changes were attributed to the minor regulatory mechanisms. This finding is valuable for understanding the gene expression order for genome evolution and fitness increase. Future studies to clarify the reason why the correlation coefficients were improved in reverse direction (**Figure 4**) are important to understand the genome evolution and growth fitness of the cells. Moreover, the regulatory networks showed that the improved correlations in expression specifically attributed to genome reduction or steady state reflected the varied mechanisms of gene expression re-ordering. The only transcriptional network that was specifically influenced by steady state was *rpoN*, a sigma factor (σ54) participating in global transcriptional regulation (Buck et al., 2000). The increased correlation of the expression of *rpoN*’s regulatees to growth rate suggested that the *rpoN* network was highly coordinated to the exponential growing phase independent of culture conditions. In contrast, the σ70 transcription factor (*rpoD*) presented similar regulatory functions (Campbell et al., 2008), as its regulatees were re-ordered in a growth-coordinated manner only due to genome reduction. Such intriguing dissimilarity might reflect the functional differentiation of *rpoN* and *rpoD* in response to the physiological and genomic fluctuations, respectively.

The present study provides a first trial of addressing whether genome reduction and cellular state interrupt the order of global gene expression. The multilevel and comparative analyses successfully observes both the difference and the similarity in the transcriptome reorganization mediated by the genome size and the cellular state. The results strongly imply that the redundant genomic sequences participated in ordering the gene expression and contributed to growth fitness. The changes attributed to genome reduction were more significant than those mediated by the cellular state. These results indicate that the genome evolution by incorporation of foreign genetic sequences might have played an essential role in global reorganization of gene expression to reach an adaptive transcriptome. So far, these conclusions are drawn upon *E. coli* solely, due to the limitation in the qualified data sets. Nevertheless, these findings strongly imply that the phenotypic profile-associated genetic information is highly essential for the quantitative understanding of a growing cell population. To reach a common opinion, highly systematic analyses across the bacterial species are required. It could be achieved by both the genetic construction the computational analyses, and the theoretical simulations. The effort has been made by systematic acquisition of the large data sets connecting the genotypes to the phenotypes, such as, the growth capacity of the single-gene knockout strains (Baba et al., 2006; Takeuchi et al., 2014), the growth profiles associated reduced genomes (Kurokawa et al., 2016) and transcriptome (Ying et al., 2016), the informative phenotypes of bacterial mutants under diverse environments (Deutschbauer et al., 2014; Wetmore et al., 2015), etc. These studies exploring the relationships among the global parameters, e.g., genome, transcriptome, and fitness, are to understand the living cell in a global view (Nichols et al., 2011). The present study offers an alternative viewpoint on the relationships among these global parameters, to illustrate an overall feature of the living cells. Further interdisciplinary studies with the big data of the global parameters should be challenged for the prediction of the phenotypes (e.g., fitness) upon the genetic and/or environmental patterns.

## Materials and Methods

### Strains, Culture Conditions, and Growth Information

Fifty-six different experimental conditions were collected from published reports (Matsumoto et al., 2013; Murakami et al., 2015; Yama et al., 2015; Ying et al., 2015, 2016) and analyzed in the present study. These conditions comprised the following *E. coli* strains: the MG1655 and DH1 strains carrying the full-length genomes and the MDS42 strains carrying the reduced genome, which has not only prophages and ISs deleted but also genes encoding membrane associate proteins, such as fimbriae and flagella (Posfai et al., 2006). These strains were grown under varied conditions, which were described in detail previously (Matsumoto et al., 2013; Murakami et al., 2015; Yama et al., 2015; Ying et al., 2015, 2016). These conditions, including genotypes, media, temperatures, etc., were summarized in **Supplementary Table S1**. All 56 conditions were categorized into various types, regular condition, high temperature, osmotic pressure, and starvation, according to the growth environments. The growth phases were also differentiated. The growth rates of the exponential growing phase and the late responsive phase (2 h after amino acid depletion) were calculated; and those of the early responsive states, such as, 5–30 min after heat shock or amino acid depletion, were defined as zero, because the cells paused to grow. All this information (56 different experimental conditions) was carefully re-confirmed and categorized according to two standards, namely genome size (full length or reduced) and cellular state (steady or unsteady), as summarized in **Supplementary Table S1**. The growth rates (h^-1^) were cited or calculated according to previously published reports (Matsumoto et al., 2013; Murakami et al., 2015; Yama et al., 2015; Ying et al., 2015, 2016).

### Expression Data Collection and Mining

A total of 168 microarray data sets (56 conditional variations with biological repetition), which were all based on the platform of EcFS, were obtained from published studies. These microarray data sets were all associated with growth information, such as growth rates, as previously summarized. Microarray raw data sets assigned with the GEO access numbers of GSE33212, GSE49296, GSE55719, GSE52770, and GSE61749 were first cut with a statistic threshold according to the *p*-values of the calculated mRNA concentrations for each gene. If any of the 168 measurements (mRNA concentrations) of the gene had a significant *p*-value (*p* < 0.05), then the gene remained in the data sets. Thus, relatively reliable values among the 168 data sets were used. A total of 3123 genes in the full length genomes of MG1655 and DH1 derivatives (128 data sets) as well as 2415 genes in the MDS42 reduced genome and its derivative (40 data sets) were subjected to global normalization, resulting in a common mean value (logarithmic) in all data sets. The normalized 168 data sets comprising the biological replicates (*N* = 2 to 7) were subsequently averaged, which resulted in 56 variations, including 44 of full length and 12 of reduced genomes. These 56 data sets were used for the following computational analyses.

### Computational Analyses

Transcriptome analyses were performed with R (Ihaka and Gentleman, 1996) as previously described (Yama et al., 2015; Ying et al., 2015). Cluster analysis was performed using the “hclust” clustering function in R. In this analysis, the expression data of 2349 genes common among both full length and reduced genomes were used. Distances were calculated by several different methods, including the furthest neighbor, group average and Ward’s methods, as shown in **Figure 1** and **Supplementary Figure S1**. The power-law analysis used all 3123 and 2415 genes in the full length and reduced genomes, respectively. The cumulative probability of the gene expression level (log scale) (Keller, 2005) was calculated from -1 to 2, and the regression was performed to estimate the slope, *r*. The classification of gene categories was performed in accordance with the original study (Riley et al., 2006) as previously described (Matsumoto et al., 2013; Ying et al., 2015). Gene categories comprising more than 15 genes were used in the analysis. A total of 20 and 18 gene categories were assigned in the full length and reduced genomes, respectively. Detailed information of transcriptional networks, including the regulators (i.e., sigma factors and transcriptional factors) and the downstream regulatees (i.e., the genes under the control of the corresponding regulator), were obtained from RegulonDB 8.0^1^ (Salgado et al., 2013). Only transcriptional networks comprising more than 15 regulatees were employed in the analysis. A total of 44 and 42 transcriptional networks were assigned in the full length and reduced genomes, respectively.

## Author Contributions

KY and B-WY performed the analyses. B-WY conceived the research and wrote the paper. All authors read and approved the final manuscript.

## Conflict of Interest Statement

The authors declare that the research was conducted in the absence of any commercial or financial relationships that could be construed as a potential conflict of interest.

## Footnotes

1 http://regulondb.ccg.unam.mx
