# Supplementary material for: Gene Expression Order Attributed to Genome Reduction and the Steady Cellular State in Escherichia coli
Source: Front Microbiol. 2018 Sep 20;9:2255. doi: 10.3389/fmicb.2018.02255 (PMC6158460; doi:10.3389/fmicb.2018.02255)
Supplement: Supplementary file 2 [file Data_Sheet_1.PDF]

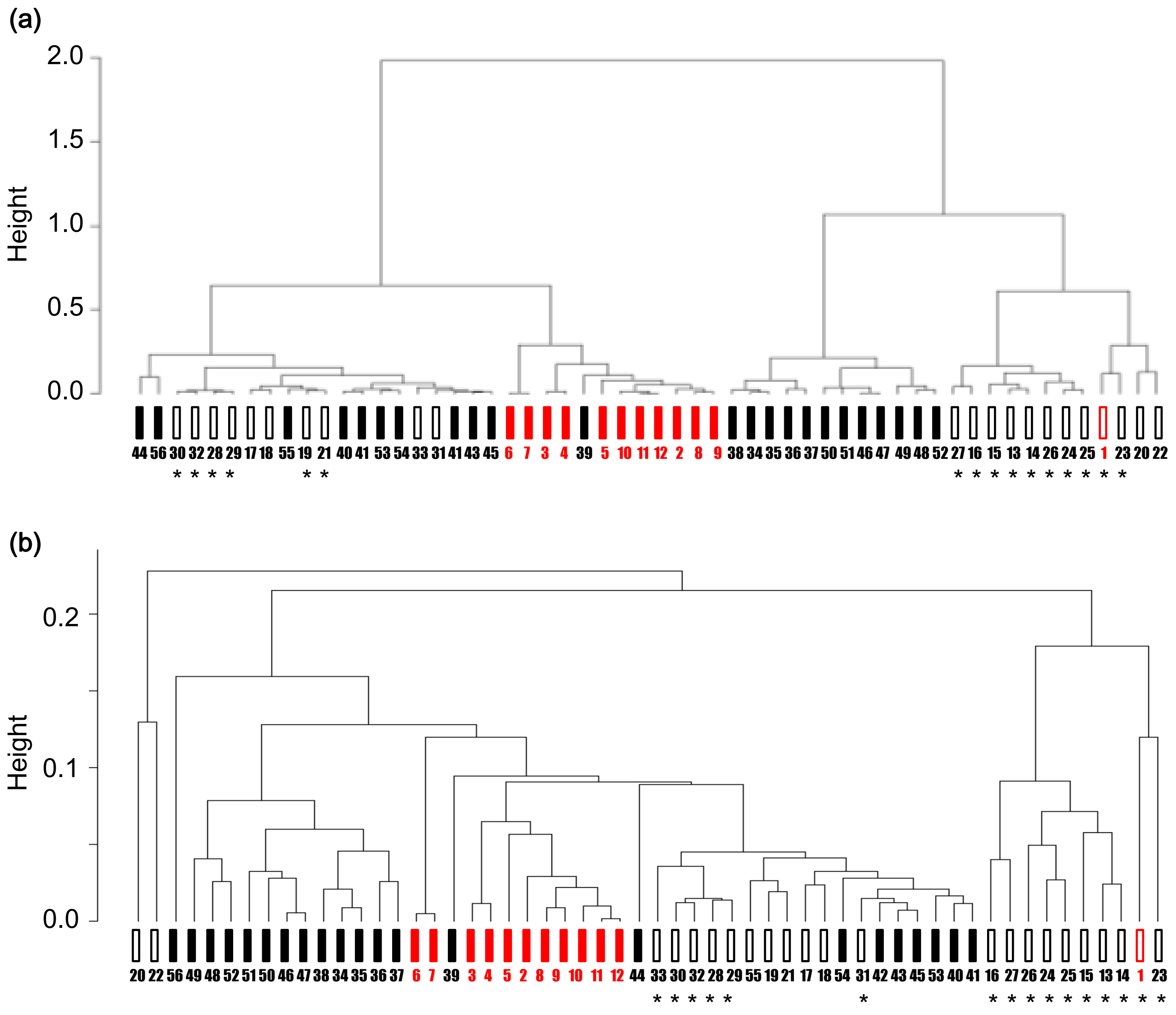

Figure S1 Varied dendrograms of 56 transcriptomes. Clustering analyses of gene expression were performed using the Ward's method (A) and the group average method (B), respectively. Filled and open bars represent the steady (exponentially growing states) and unsteady (responsive or intermediate states) cellular states, respectively. Black and red indicate the full length and reduced genomes, respectively. Asterisks indicate the unsteady states of zero growth. Nos. 1-56 correspond to 56 variations described in Table S1.

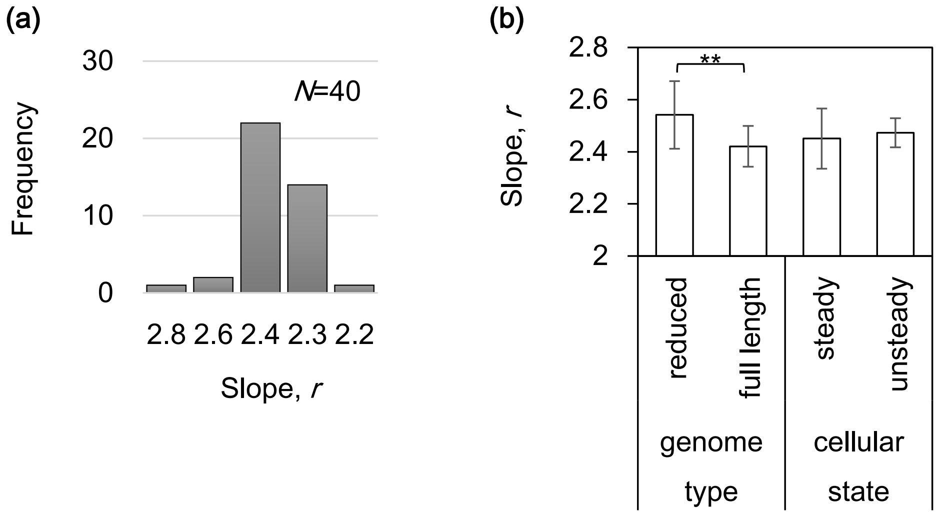

Figure S2 The power law in gene expression without zero growth data sets. (**a**) Histogram of the slopes. The slopes (*r*) of 40 distributions (transcriptomes) were calculated and summarized in a histogram with a bin of 0.2. (**b**) Differentiated slopes in reduced genomes. The slopes of 40 distributions were averaged according to the categories of either genome type (full length and reduced) or cellular state (steady and unsteady), respectively. Statistical significance is indicated by a dual asterisks (P<0.01).

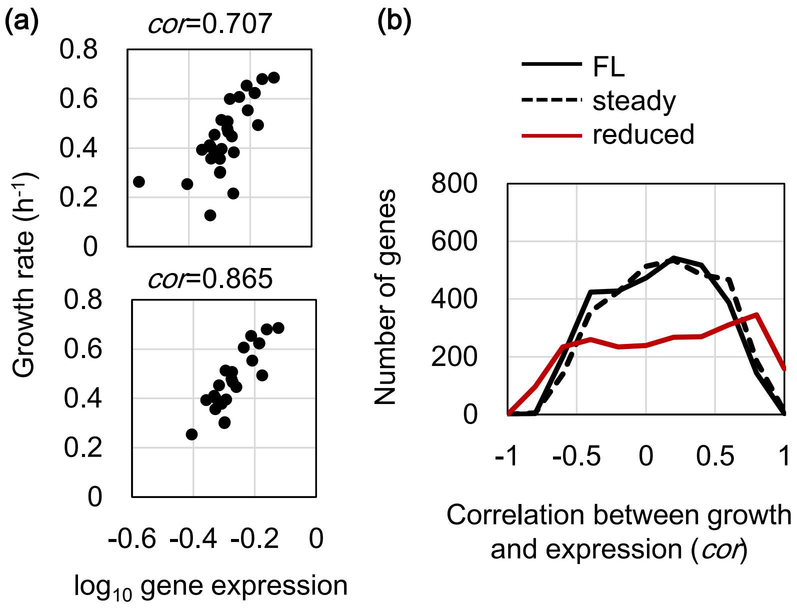

Figure S3 Distribution of the correlation coefficients between gene expression and growth rate. (**a**) Correlation between gene expression level and growth rate. The expression levels (mRNA concentrations in log scale) of a single gene in varied conditions were plotted against the growth rates of the corresponding conditions. The upper and bottom panels show all 29 conditions and 23 steady state conditions of *mrdB* (JW0629) in the full length genomes. The correlation coefficients are indicated. (**b**) Changes in distributions of the correlation coefficients. A total of 3213 and 2415 correlation coefficients were calculated in the full length and reduced genomes as shown in (a). The numbers of the genes were counted within a bin of 0.1 for the correlation coefficient. Black solid, broken, and red lines indicate the distributions formed by 29 full length genomes, 23 steady growth of full length genome, and 12 reduced genomes, respectively. The grey line stands for the distribution formed by 29 full length genomes, in which the genes deleted in the reduced genomes were excluded from the analysis.

.

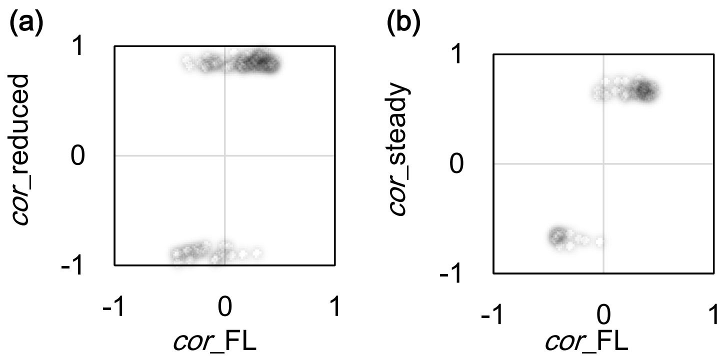

Figure S4 Genes of improved correlations. (**a**) Genes of improved correlations due to genome reduction. A total of 100 genes randomly expressed (P>0.01) in full length genomes changed to growth rate-coordinated expression (P<0.001) in reduced genomes. (**b**) Genes of improved correlations mediated by the steady cellular state. A total of 57 genes randomly expressed (P>0.01) in all 29 conditions changed to growth rate-coordinated expression (P<0.001) in 23 steady growth conditions.

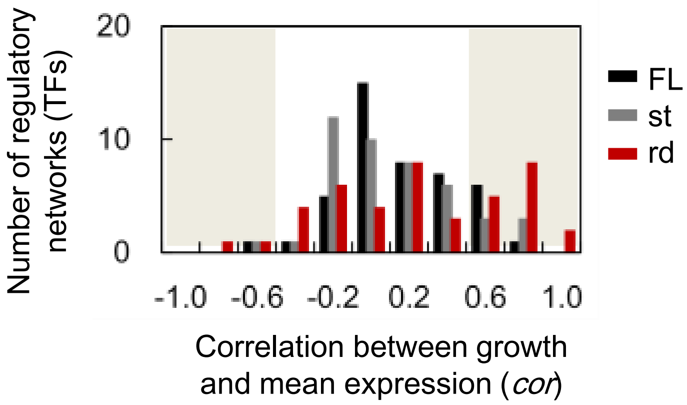

Figure S5 Histograms of the correlation coefficients without the data sets of zero growth. A total of 44 and 42 correlation coefficients (regulatory networks) were evaluated in the full length and reduced genomes. The numbers of the regulatory networks were counted within a bin of 0.2 for the correlation coefficient. Black, grey, and red bars indicate the distributions formed by 29 full length genomes (FL), 23 steady growth of full length genome (st), and 11 reduced genomes (rd), respectively. The shadowed region indicates the statistical significance (p<0.05).

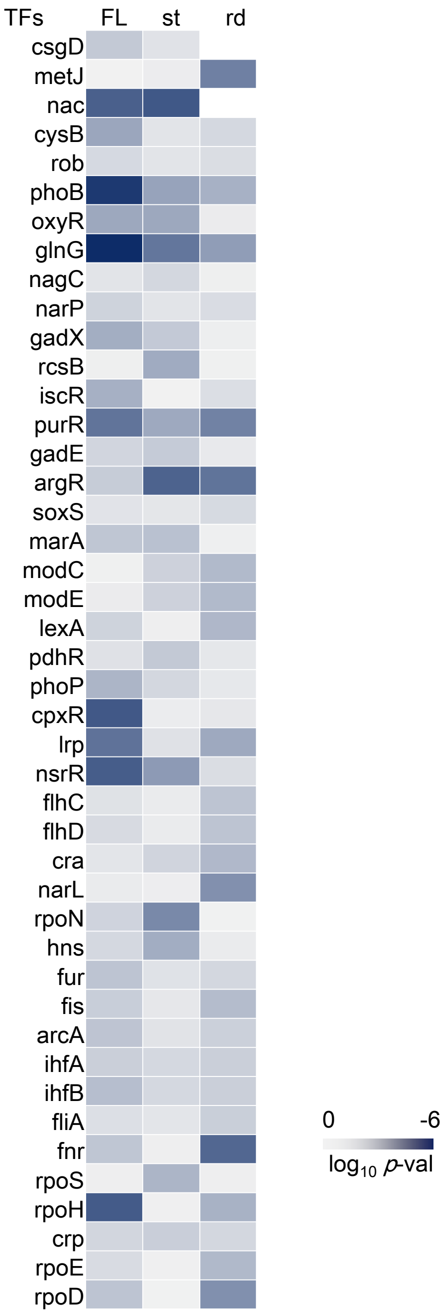

Figure S6 Correlations between the growth rates and the expression of regulatory networks. (**a**) Changes in distributions of the correlation coefficients. A total of 44 and 42 correlation coefficients (regulatory networks) were evaluated in the full length and reduced genomes, respectively, as shown in Figure 5a. The numbers of the regulatory networks were counted within a bin of 0.2 for the correlation coefficient. Black solid, broken, and red lines indicate the distributions formed by 44 full length genomes, 22 steady cellular state of full length genome, and 12 reduced genomes, respectively. (**b**) Significance of correlation coefficients. A total of 44 regulatory networks are shown. FL, st and rd represent the correlation coefficients in a total 44 full length genomes, 22 steady growth of full length genomes and 12 reduced genomes, respectively. Graduation from dark blue to light grey indicates the statistical significance of the correlation coefficients in log-scaled *p* values.
